# Camptothecin nanocolloids based on N,N,N-trimethyl chitosan: Efficient suppression of growth of multiple myeloma in a murine model

**DOI:** 10.3892/or.2012.1635

**Published:** 2012-01-12

**Authors:** ZHENGGUANG LI, XINGYI LI, ZHIXING CAO, YOUZHI XU, HONGJUN LIN, YINGLAN ZHAO, YUQUAN WEI, ZHIYONG QIAN

**Affiliations:** 1 Department of Oncology, The Third Affiliated Hospital of Soochow University, Changzhou 213003; 2 State Key Laboratory of Biotherapy and Cancer Center, West China Hospital, West China Medical School, Sichuan University, Chengdu 610041, P.R. China

**Keywords:** camptothecin, N,N,N-trimethyl chitosan, myeloma, antitumor

## Abstract

Camptothecin (CPT) exhibits very strong antitumor effects by inhibiting the activity of DNA topoisomerase I, but its application is greatly limited due to its low solubility and the instability of the active lactone form. To overcome these shortcomings, in the present study, we prepared novel camptothecin nanocolloids based on N,N,N-trimethyl chitosan (CPT-TMC) to efficiently and safely administer CPT systemically. Herein, we investigated the antitumor activity of CPT-TMC against a murine Balb/c myeloma model. Our results showed that CPT-TMC more effectively inhibited tumor growth and prolonged survival time than CPT *in vivo*, but no statistical difference was observed *in vitro* between CPT-TMC and CPT. These findings suggest that N,N,N-trimethyl chitosan could increase the stability and the antitumor effect of CPT and CPT-TMC is a potential approach for the effective treatment of multiple myeloma.

## Introduction

Multiple myeloma (MM), a malignant plasma cell disorder, accounts for about 10% of all hematological cancer cases (1,2). The annual incidence of MM varies between 1 to 5 cases per 100,000 persons worldwide (3). As the second most frequent malignancy of the blood in the USA after non-Hodgkin’s disease, about 19,900 new cases of MM and 10,790 deaths occurred in the United States (4). Since the introduction of alkylating agents and melphalan in the 1960s, the median survival of patients with MM has improved (5), but the treatment outcome is far from satisfactory, and novel drugs are in urgent demand to better combat this malignancy.

Camptothecin (CPT) was first isolated by Monroe E. Wall and Mansukh C. Wani in 1958 from extracts of *Camptotheca acuminata*, a deciduous tree native to China and Tibet, which has been extensively used in traditional Chinese medicine (6). CPT represents an important class of agents useful in the treatment of cancer, which show a broad spectrum of antitumor activity, including lung, ovarian, breast, pancreas, stomach and leukemia (7–11). CPT exhibits antitumor effects by inhibiting the activity of DNA topoisomerase I, which is required for replication and transcription of the cell cycle and stabilization of the DNA-topoisomerase complex, thereby resulting in single-strand DNA breaks to induce the apoptosis of cancer cells (12–15). Cancer cells often overexpress topoisomerase 1 (Topo-1), and they are usually more susceptible to CPT than normal cells (16). It is suggested that CPT may be a promising anticancer agent that warrants further investigation.

**Figure 1:**
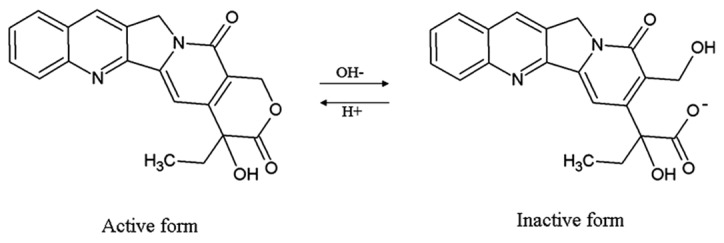
Camptothecin structure and equilibrium between the active lactone form and the inactive carboxylate form.

However, there are some drawbacks of CPT significantly restricting its clinical use. CPT, similarly to a number of other potent anticancer agents of plant origin, is extremely water insoluble and can only be solubilized in dimethylsulfoxide (DMSO), dichloromethane: methanol (1:1) (v:v) and chloroform: methanol (4:1) (v:v). The serious side effects of co-solvents and bioavailability problems have hampered the usage of CPT *in vivo* (13). The lactone ring in CPT plays an important role in the drug’s biological activity but it opens at a physiological or higher pH values, making this drug much less active and highly toxic, and precluding its clinical use (Fig. 1) (17).

To overcome these drawbacks of aqueous solubility and stability, two strategies have been introduced. One is to synthesize water-soluble analogues, pro-drugs, and derivatives of CPT. Thus far many compounds have been reported, such as CPT-11, SN-38 and DX-8951f (18–22). However, these compounds are not stable enough *in vivo* and have lower activity than CPT (23). Alternatively, the development of adequate drug carrier systems to improve the solubility and stability of CPT is gaining attention. There are many reports about utilization of CPT in cancer therapy by using drug delivery systems, such as liposomes, polymer micelles, microemulsions, and microspheres (17,24–28). Unfortunately, these carriers are unsatisfactory due to the poor biocompatibility and biodegradability.

Chitosan is an aminoglucopyran composed of N-acetylglucosamine and glucosamine residues and has excellent properties, such as biocompatibility, biodegradability, and is nontoxic (29). However, the application of the polymer in medicine as a drug carrier *in vivo* is difficult to be achieved due to its insolubility. Thus, there is a need for chitosan derivatives with increased solubility, especially at neutral pH values, to aid in the delivery of therapeutic compounds (30,31). Trimethyl chitosan has been proven to be a derivative of chitosan with superior solubility compared to chitosan (32). N-Methylated chitosan with the hydrophilic groups N^+^(CH_3_)_3_ and the hydrophobic groups N(CH_3_)_2_ is amphiphilic and water-soluble in character at physiological pH and can be self-assembled to vesicles. N-Methylated chitosan has been previously used as a carrier for the delivery of small drug molecules due to its properties (33–35).

In the present study, we chose N,N,N-trimethyl chitosan (TMC) as a carrier to encapsulate CPT and to overcome its drawbacks. The anticancer efficacy of CPT encapsulated with N,N,N-trimethyl chitosan (CPT-TMC) was examined *in vivo* and *in vitro*.

## Materials and methods

### Materials

N,N,N-Trimethylated chitosan (TMC), camptothecin (CPT), propidium iodide (PI), 3-(4,5-dimethylthiazol-2-yl)-2,5-diphenyltetrazolium bromide (MTT), RNase A and dimethyl sulfoxide (DMSO) were purchased from Sigma Chemical Co. (St. Louis, MO). All the chemicals employed in this study were of analytical purity and of culture grade. The *in situ* cell death detection kit was purchased from Roche Co. (Promega, Madison, WI).

### Cell culture and tumor model

The murine Balb/c myeloma cell line MPC-11 was purchased from the American Type Culture Collection (ATCC, Manassas, VA) and cells were grown in RPMI-1640 (Life Technologies, Bedford, MA) containing 10% heat-inactivated FCS, 100 units/ml penicillin, and 100 units/ml streptomycin in a humid chamber at 37˚C under 5% CO_2_.

The MPC-11 tumor model was established in 8-week-old female BALB/c mice. Briefly, these BALB/c mice were inoculated subcutaneously with MPC-11 cells (2×10^5^) in the dorsal area. All these mice were purchased from the Sichuan University Animal Center (Sichuan, Chengdu, China). All studies involving mice were approved by the Institute’s Animal Care and Use Committee.

### Preparation of CPT-TMC nanocolloid

According to a previous study (36), CPT-TMC nanocolloid was successfully prepared by a combination of microprecipitation and sonication. Briefly, 6 mg/ml of CPT was first prepared by dissolving 30 mg CPT into 5 ml DMSO. Then TMC was dissolved in water solution at the concentration of 5 mg/ml. Subsequently, 0.1 ml of CPT solution was added in a dropwise fusion into 2 ml of TMC solution at 4˚C. The obtained colloid solution was ultrasonicated for 10 min keeping the temperature at 4˚C. Finally, the colloid solution was dialyzed against water using a membrane with a molecular weight cut-off of 8,000–14,000 (Solarbio, China). After dialysis for 3 days, the solution was centrifuged at 10,000 × g for 10 min to remove insoluble CPT. The amount of CPT in the TMC solution was measured by HPLC.

### In vitro cytotoxicity assay

The growth-inhibitory activity of CPT-TMC on the MPC-11 cell lines was evaluated by MTT assay. Briefly, the MPC-11 cells (4–5×10^3^) were seeded in 96-well plates and cultured for 24 h, followed by exposure to various doses of free CPT or CPT-TMC with equivalent doses of CPT for 48 h. A volume of 10 μl of 10 mg/ml MTT was added per well and incubated for another 4 h at 37˚C, then the supernatant fluid was removed and 150 μl/well DMSO was added for 15–20 min. The light absorption values (OD) were measured at 570 nm with the SpectraMAX M5 microplate spectrophotometer (Molecular Devices). The viability of cells was measured by the absorbance at 570 nm.

To assess the effect of CPT-TMC on cell apoptosis and the cell cycle, flow cytometric analysis was performed to measure the percentage of sub-G1 cells after PI staining in hypotonic buffer as previously described (37,38). Briefly, cells were suspended in 1 ml hypotonic fluorochrome solution containing 50 μg/ml PI in 0.1% sodium citrate plus 0.1% Triton X-100 and the cells were analyzed by a flow cytometer (ESP Elite, Beckman-Coulter, Miami, FL). Apoptotic cells appeared in the cell cycle distribution as cells with a DNA content of less than that of G1 cells and were estimated with the Listmode software.

For morphological analysis, the cells were fixed using 70% of ethanol following rinsing with PBS. Morphological analysis of apoptosis was performed after staining with PI (1 μg/ml, in PBS) under fluorescence microscopy (Axiovert 200, Zeiss, Germany) or under light microscopy without staining.

The pattern of DNA cleavage was analyzed as previously described (39). Briefly, cells (3×10^6^) were lysed with 0.5 ml lysis buffer [5 mM Tris-HCl (pH 8.0), 0.25% Nonidet P-40, and 1 mM EDTA], followed by the addition of RNase A at a final concentration of 200 μg/ml, and incubated for 1 h at 37˚C. Cells were then treated with 300 μg/ml proteinase K for an additional 1 h at 37˚C. After addition of 4 μl loading buffer, 20 μl samples in each lane were subjected to electrophoresis on a 1.5% agarose gel at 50 V for 3 h. DNA was stained with ethidium bromide.

### In vivo antitumor activity

MPC-11-bearing Balb/c mice were coded and divided into four groups (n=10 for each group). Each group was respectively treated with intravenous injections of CPT-TMC (2.5 mg/kg), CPT (2.5 mg/kg), TMC (25 mg/kg) or 0.9% NS. Treatment was initiated when the tumor volume was 90 mm^3^. Treatments were given every 3 days for 15 days and survival time and tumor volumes were observed. Tumor size was determined by caliper measurement of the largest and perpendicular diameters every three days. Tumor volumes were calculated according to the formula: V=axb^2^×0.52, where a is the largest superficial diameter and b is the smallest superficial diameter. The mice were sacrificed when they became moribund. The date of sacrifice was recorded to calculate the survival time. For further investigation, tumors tissues were excised and fixed in 10% formalin.

### Apoptosis analysis in tumor tissues

Tumor tissues in paraffin blocks were cut into sections of 3–5 μm thickness. Apoptosis analysis was performed by terminal deoxynucleotidyl transferase-mediated dUTP nick-end labeling staining using the DeadEnd™ Fluorometric TUNEL system (Promega) following the manufacturer’s protocol. Four equal-sized fields from the tissue sections were randomly chosen and analyzed. The positive-stained cells were visualized and analyzed under a fluorescence microscope (Olympus, BX60). The apoptotic index was calculated as a ratio of the positive cell number to the total tumor cell number based on the mean value from four high-power fields.

### Statistical analysis

Data were assayed by ANOVA and the Student’s t-test. For the survival time, Kaplan-Meier curves were established for each group, and the survivals were compared by the log-rank test. All data were presented as mean ± SD. Experiments were performed at least in duplicate. All data were analyzed using the SPSS software (SPSS for Windows, version 17.0; SPSS, Chicago, IL). In all statistical analyses, P<0.05 denoted significant differences.

## Results

### Inhibition of cell viability

**Figure 2:**
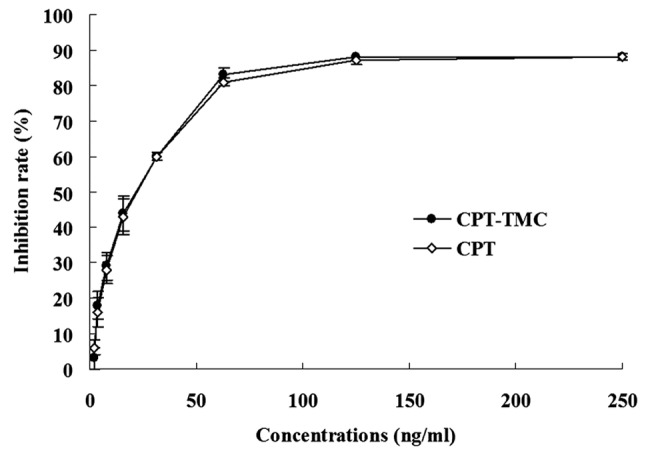
Dose-dependent inhibition of the viability of MPC-11 cells treated with CPT-TMC and CPT. MPC-11 cells were treated with various doses of CPT-TMC and CPT for 48 h. During the assays, the CPT dose in the CPT-TMC was always the same as that of free CPT. Cell viability was detected by MTT assays. Data are expressed as mean ± SD. No significant difference was observed between the CPT-TMC and the CPT group (P>0.05).

According to the results of the MTT assay, exposure to CPT-TMC and CPT for 48 h, significantly inhibited the cell viability of MPC-11 cells. The results directly suggest that CPT-TMC and CPT inhibited cell viability in a concentration-dependent manner. No statistical difference was observed between the CPT-TMC and the CPT group (P>0.05, Fig. 2).

### In vivo antitumor activity

**Figure 3:**
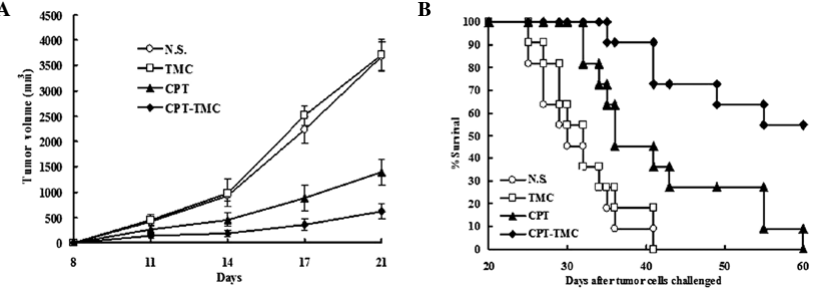
Tumor suppression and survival advantage in mice. (A) The CPT-TMC group shows significant differences in the tumor volume compared with NS, TMC and the CPT groups (P<0.05) from day 11. (B) Survival time was significantly prolonged by CPT-TMC compared with the other three control groups (log-rank test, P<0.05). Data shown are mean ± SD.

MPC-11-bearing Balb/c mice were treated with CPT-TMC (2.5 mg/kg), CPT (2.5 mg/kg), TMC (25 mg/kg) and NS, respectively. No differences were observed between the TMC group and the NS group in terms of tumor growth (P>0.05), and it was proven that TMC did not have antitumor activity. On the contrary, CPT and CPT-TMC were found to have antitumor efficiency in inhibiting tumor progress. The inhibition rate of the tumor volume treated with CPT-TMC was 83% compared with the NS group (P<0.05). It was interesting that, as shown in Fig. 3A, the antitumor efficiency of CPT-TMC was better than that of CPT by 55% (P<0.05). However, no complete reaction was found in all these groups.

To further investigate the antitumor effects of CPT-TMC *in vivo*, we assayed the lifespan of the mice. Our results show that the groups treated with NS survived 31 days on average, and there was not significant difference between the TMC and NS groups (P>0.05). In contrast, systemic therapy with CPT-TMC significantly prolonged the survival time vs. the NS (P<0.05) and CPT group (P<0.05). When the study was terminated at 60 days after inoculation, more than half of the animals in the CPT-TMC group had survived (Fig. 3B). These data indicate that the animals significantly benefited from CPT-TMC treatment.

### Induction of cell apoptosis in vitro and in vivo

The effect of apoptosis induction of CPT-TMC *in vitro* was measured by flow cytometric analysis, DNA ladder, morphological analysis and the TUNEL assay.

**Figure 4:**
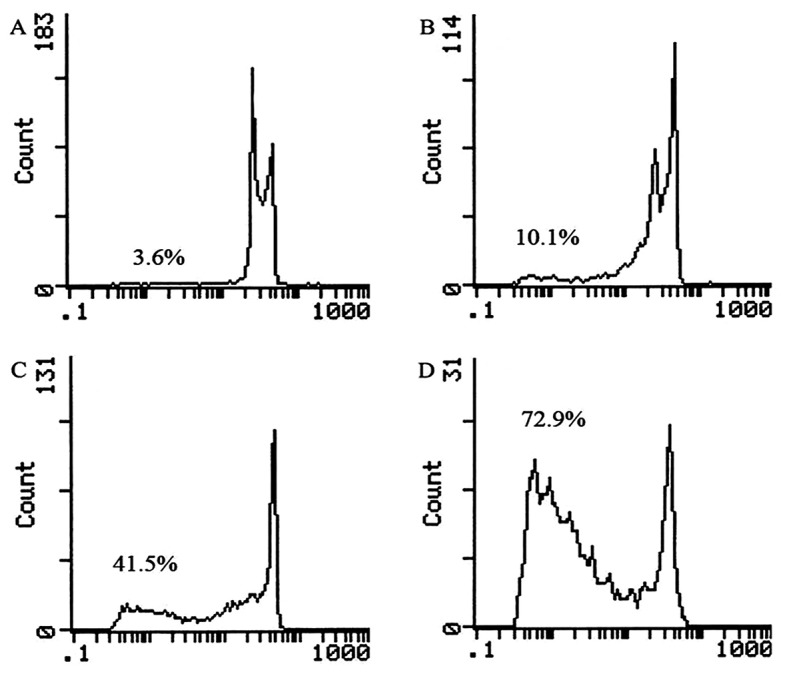
Representative DNA fluorescence histograms of propidium iodide (PI)-stained cells. MPC-11 cells were (A) untreated or treated with various doses of CPT-TMC [(B) 12.5 ng/ml, (C) 25 ng/ml, (D) 50 ng/ml] for 48 h. The cells in the sub-G1 phase were considered as apoptotic cells. The apoptosis rates in non-treated and CPT-TMC-treated cells were (A) 3.6%, (B) 10.1%, (C) 41.5%, (D) 72.9% as assessed by flow cytometry.

By the use of flow cytometry, we could assess the number of sub-G1 cells, which can be used to indirectly estimate the number of apoptotic cells. The results obtained with flow cytometry strongly show that the CPT-TMC treatment led to MPC-11 cell death by inducing apoptosis in a dose-dependent manner. After exposure to CPT-TMC for 48 h, apoptosis could be observed at 12.5 ng/ml. The increased number of apoptotic cells was detected and the apoptosis rate reached 72.9% at 50 ng/ml (Fig. 4).

**Figure 5:**
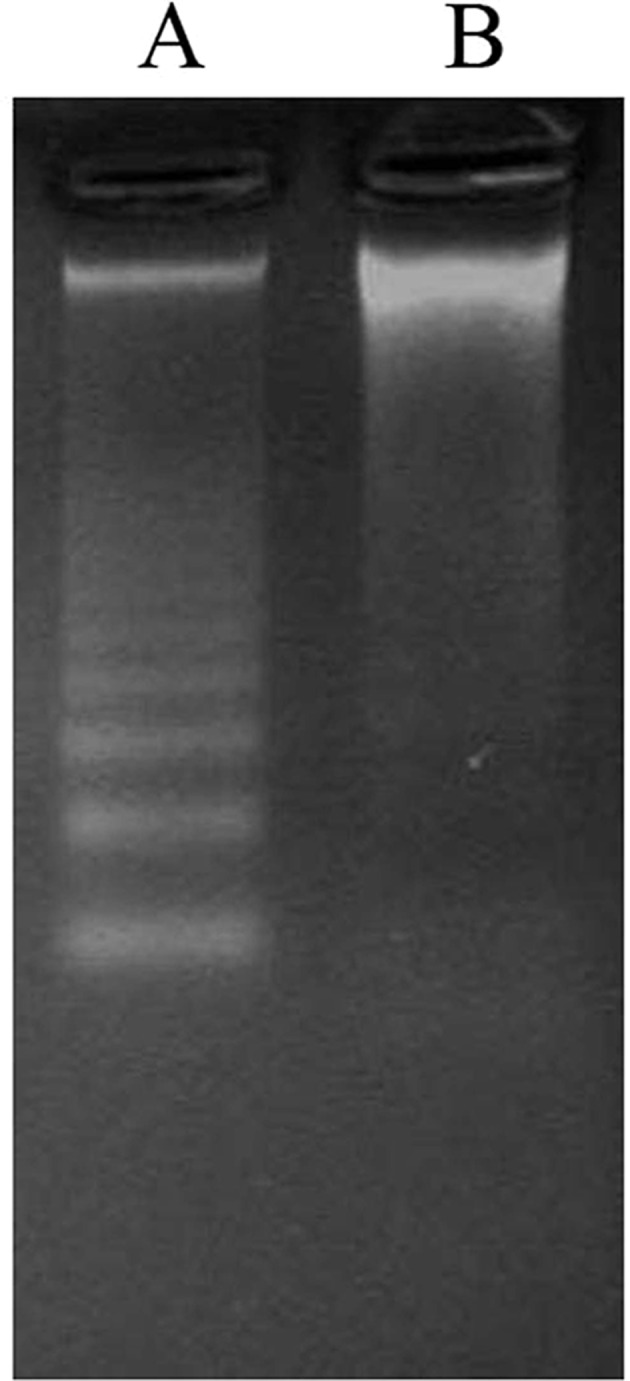
Agarose gel electrophoretic patterns of DNA. Agarose gel electrophoretic patterns of DNA isolated from MPC-11 cells (A) treated and (B) untreated with CPT-TMC (50 ng/ml) for 24 h.

To further confirm the apoptosis induction effect of CPT-TMC, the pattern of DNA cleavage was analyzed after treatment with CPT-TMC demonstrated a ladder-like pattern of DNA fragments consisting of multiples of approximately 180–200 base pairs, consistent with internucleosomal DNA fragmentation (Fig. 5).

**Figure 6:**
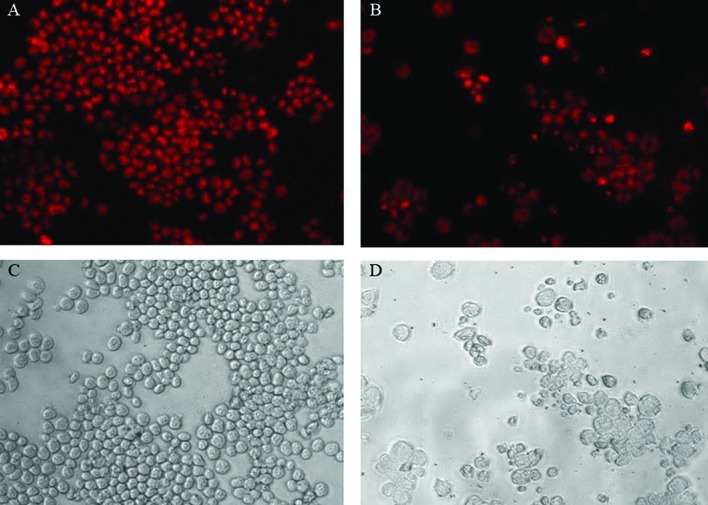
Morphological changes induced by CPT-TMC. Bright-field microscopy images (C and D) and fluorescence microscopic appearance of PI-stained nuclei (A and B) of MPC-11 cells untreated or treated with CPT-TMC (25 ng/ml) for 48 h (original magnification, ×400).

Treatment with CPT-TMC also resulted in morphological changes consistent with apoptosis. Compared with the control, cells treated with CPT-TMC for 48 h were vacuolated, had shrunk and gradually showed increased membrane blebbing as the CPT-TMC concentration increased. After PI staining, the morphological changes were also characteristic of apoptosis: brightly red, condensed nuclei (intact or fragmented) were observed by fluorescence microscopy (Fig. 6).

**Figure 7:**
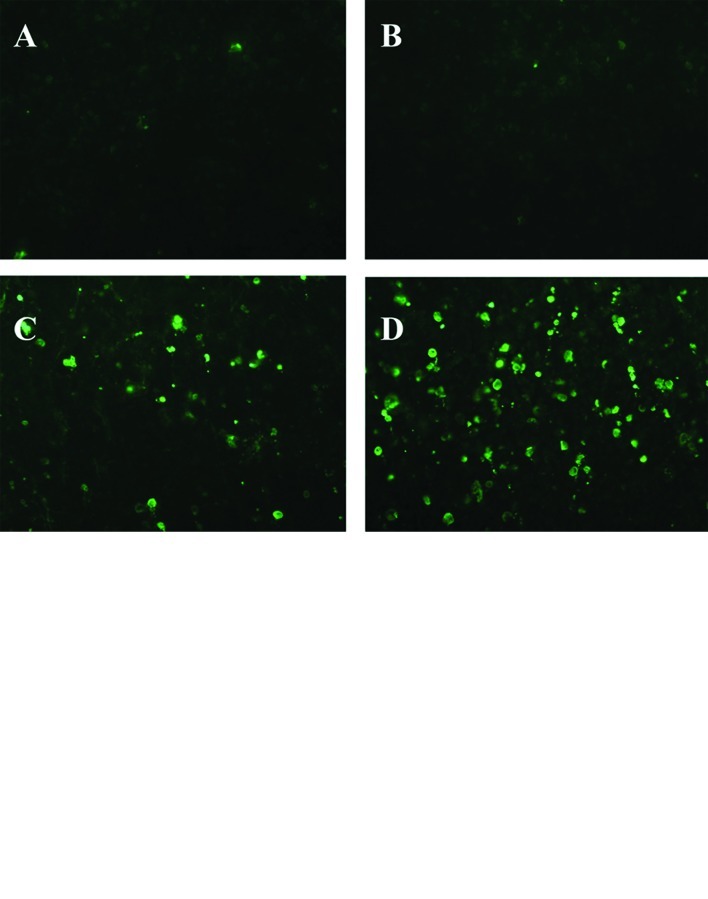
TUNEL assay. (A) Only a few positive nuclei were observed in tumor tissues of NS and TMC controls (A and B), and there were many more positive nuclei in the CPT-TMC group than in the CPT group (C and D). (B) Percent apoptosis in each group revealed that CPT-TMC exhibited a more effective induction of apoptosis compared with the other three controls. Values are presented as the mean ± SD. (^*^P<0.05).

To investigate the apoptosis induction effect of CPT-TMC *in vivo*, the tumor tissues were subjected to terminal deoxynucleotidyl transferase-mediated dUTP nick-end labeling assays for respective determination of the apoptotic index. The results suggest that there were almost no TUNEL-positive nuclei in the NS and TMC groups. Both the CPT group and the CPT-TMC group had a higher apoptosis rate of tumor cells compared with the NS control (P<0.05). The apoptosis rate of the CPT-TMC group was much higher than that of the CPT group (P<0.05) (Fig. 7).

## Discussion

Multiple myeloma (MM) is a chronic hematological disease affecting terminally differentiated B cells, for which there is currently no cure. In the last decades, there is a noticeable improvement in the treatment of MM due to the introduction of new therapeutic strategies and new agents, such as ASCT (autologous stem cell transplantation), thalidomide, bortezomib, and lenalidomide (40). But in fact, the median survival is 4.4–7.1 years in spite of all available therapies for relapse and drug resistance (41,42). For this reason, searching for new and more effective agents is necessary.

Camptothecin (CPT), a plant alkaloid, is a potent antitumor agent, which acts by inhibiting the nuclear enzyme topoisomerase I and inhibits the growth of a wide range of tumors. Some analogues of CPT, such as topotecan, have been ultilized as chemotherapy agent in MM (43–45). However, there is limited information of CPT itself in MM, because the major drawbacks of the drug, water insolubility and lactone instability, hamper its medical use. To overcome these drawbacks, some delivery systems were developed to increase the solubility and the stability of the drug, such as liposome and polymeric micelles (17,46). But all these approaches are far from satisfactory due to poor biocompatibility, biodegradability, or bioadhesivity. Therefore, we studied the effect of CPT on murine multiple myeloma cells MPC-11 *in vitro* and *in vivo* and attempted to increase its solubility and stability.

Chitin, a linear cationic heteropolymer of randomly distributed N-acetylglucosamine and glucosamine residues, is one of the most abundant polysaccharides in nature and is mostly derived from the exoskeleton of crustaceans (47). Chitosan, a polymer obtained by deacetylation of chitin is widely studied for its pharmaceutical and non-pharmaceutical applications. For example, chitosan has been extensively evaluated for its mucoadhesive and absorption enhancement properties. But chitosan is not soluble in a medium except below pH 5.6 and this property limits its use as a drug delivery system. Therefore, there is a need for chitosan derivatives with increased solubility at physiological pH values. N,N,N-Trimethyl chitosan (TMC) is a promising derivativ. TMC is soluble either in an acidic, basic or neutral medium (pH range 1–9 up to 10% w/v concentration) and has mucoadhesive and permeation enhancement properties like native chitosan (48,49). It has been reported that TMC could enhance the transport of small compounds, large molecules, peptide drugs and DNA and has shown promising results as a drug delivery agent as well as a DNA delivery agent (50). Hence, TMC was selected as a carrier to delivery CPT which is insoluble in water.

In the present study, we investigated the antitumor effect of CPT on the murine myeloma cell line MPC-11 and we demonstrated that CPT inhibited the growth of MPC-11 cells *in vitro* and *in vivo*. To further improve the antitumor activity, we chose TMC as a carrier to encapsulate and deliver CPT (CPT-TMC). The results of the MTT assay showed that both CPT and CPT-TMC significantly inhibited the growth of MPC-11 cells *in vitro* and there was a significant difference between their effects. TMC itself was not cytotoxic and TMC could not improve the antitumor activity of CPT *in vitro*. However, interestingly, the results of *in vivo* assays were different from those of *in vitro* assays. Compared with CPT, in murine models, we demonstrated that CPT-TMC more efficiently suppressed tumor growth in murine models. Moreover, the TUNEL assay showed a significant increase of the apoptotic index in the CPT-TMC group. A further study showed that the survival time of animals treated with CPT-TMC was significantly prolonged compared with the NS, TMC and CPT groups. According to these results, it was suggested that the TMC delivery system efficiently improved the antitumor activity *in vivo*, but not *in vitro*. The mechanism of the antitumor effects of CPT-TMC may be the prolonged blood circulation time or the accumulation of CPT in tumor tissue. In light of the encouraging results presented herein, delineation of the potential chemotherapeutic effects of CPT-TMC and its precise mechanism of actions warrants further investigation.

In conclusion, we demonstrated that CPT has powerful antitumor activity through induing apoptosis in murine multiple myeloma models, and that the TMC delivery system can efficiently improve the effects of CPT. Thereby, our finding may provide a strategy to permit utilization of CPT in clinical practice. CPT-TMC may be a new effective agent to better combat multiple myeloma.
